# DNA hypomethylation of CBS promoter induced by folate deficiency is a potential noninvasive circulating biomarker for colorectal adenocarcinomas

**DOI:** 10.18632/oncotarget.17988

**Published:** 2017-05-18

**Authors:** Geng Xue, Chao-Jing Lu, Shu-Jun Pan, Yin-Ling Zhang, Hui Miao, Shi Shan, Xiao-Ting Zhu, Yi Zhang

**Affiliations:** ^1^ Department of Medical Genetics, College of Basic Medical Sciences, Second Military Medical University, Shanghai 200433, China; ^2^ Department of Thoracic Surgery, Changhai Hospital, Second Military Medical University, Shanghai 200433, China; ^3^ Department of Medical Administration, Hangzhou Sanatorium of People's Liberation Army, Hangzhou 310007, China

**Keywords:** DNA methylation, colorectal cancer, folate, CBS, cell-free DNA

## Abstract

Aberrant DNA methylation patterns, which induced by folate deficiency, play important roles in tumorigenesis of colorectal cancer (CRC). Some DNA methylation alterations can also be detected in cell-free DNA (cfDNA) of patients’ plasma, making cfDNA an ideal noninvasive circulating biomarker. However, exact DNA methylation alterations induced by folate deficiency in tumorigenesis of CRC and exact potential circulating cfDNA methylation biomarker are still unclear. Therefore, DNA methylation patterns of the normal human colon mucosal epithelial cell line (NCM460), cultured with normal or low folate content, were screened and the DNA hypomethylation of cystathionine-beta-synthase (CBS) promoter was further validated *in vitro* and vivo. Then, the correlation analysis between folate level, DNA methylation alteration in promoter and expression of CBS was carried out *in vitro* and vivo. Further, the methylation patterns of CBS promoter in plasma cfDNA were detected and statistically correlated with pathological parameters and clinical outcome. Our study showed that DNA hypomethylation in CBS promoter, induced by folate deficiency, would lead to up-regulation of CBS both *in vitro* and vivo. Patients with cfDNA hypomethylation of CBS promoter in plasma were correlated with high tumor stage and poor clinical outcome. In addition, cfDNA hypomethylation of CBS promoter in plasma was shown to be an independent prognostic factor for recurrence and cancer-related death in CRC. Our results indicated that DNA hypomethylation of CBS promoter induced by folate deficiency could serve as a potential noninvasive circulating biomarker and may be helpful in developing more effective prognostic markers for CRC.

## INTRODUCTION

Colorectal cancer (CRC) is the most common gastrointestinal tract malignancy with more than 1–2 million new cases diagnosed per annum in the world [1]. Globally, the majority of cases occur in developed countries, particularly North America, Australia and Western Europe with a worldwide annual mortality of 600,000 [2]. Observational studies on people who migrate from low to high-risk countries support the evidence that causes of CRC are largely environmental compared to age or gender related and the main characteristic in these studies has been a change from a prudent diet to a Westernized diet with higher intake of energy dense foods and lowered physical activity [3, 4]. Great advances in epigenetics have been made in understanding these ecological findings and apart from genetic changes, epigenetic modifications play a major role in CRC [5, 6]. Epigenetic processes are essential in normal development and differentiation but may be misdirected and predisposed to tumorigenesis [7].

Despite significant advances, the prognosis of CRC patient is still poor owing to the lack of effective biomarkers for early diagnosis and optimal therapeutic decision making [1, 8]. The well-known epigenetic marker remains to be DNA methylation, which often occurs very early before the tumorigenesis of CRC [5, 9]. To date, many studies have evaluated DNA methylation as a valid marker for CRC predisposition [10] and unlike DNA mutations, DNA methylation that can be consistently measured, as it tends to occur in specific regions of the DNA [5, 10]. Moreover, DNA methylation alterations found in tumor cells are also reflected in circulating cell-free DNA (cfDNA) released from the tumor tissues into the blood, making cfDNA an ideal noninvasive biomarker for cancer diagnosis and prognosis [11, 12]. Therefore, detection of circulating methylated-DNA in plasma represents one of the most promising methods for early diagnosis and prognosis of CRC. However, a range of methylation alterations occur during the tumorigenesis of CRC, which would really be helpful in diagnosis and prognosis are still unclear.

As we all known, diet is a major aspect of the environment may influence DNA methylation [13] and especially interesting are nutrients, which are needed for nucleic acid and DNA synthesis and for the enzymes regulating their syntheses, for example, essential amino acids, zinc, vitamins, and so on [14, 15]. One of most strongly implicated nutrients is folate, which is a generic term for a naturally occurring family of B-group vitamins composed of an aromatic pteridine ring linked to p-aminobenzoic acid and a glutamate residue B-6 and B-12 [3, 16]. Folate is a natural constituent of foods such as green leafy vegetables, legumes, and citrus fruits [3, 17]. Folate which plays a vital role in one-carbon metabolism is crucial for DNA methylation and the most important potential mechanism for folate deficiency mediated carcinogenesis is aberrant global or regional methylation [16, 18]. An inverse relationship between folate intake and risk of CRC was demonstrated in the follow-up study where the relative risk of adenomas was 0.66 for women and 0.63 for men in those with a higher intake of folate [19]. Over the past few years, the effect of folate deficiency on DNA methylation patterns has been studied [18, 20], however, results from studies are inconsistent, with some showing hypomethylation, no change or hypermethylation.

For the present study, we identified DNA hypomethylation of CBS promoter induced by folate deficiency and validated putative role of the DNA hypomethylation of cfDNA in patients’ plasma as a potential noninvasive circulating biomarker for CRC.

## RESULTS

### Comprehensive set of aberrant methylation patterns induced by folate deficiency

The number of methylated CpG islands were 3073 in normal human colon mucosal epithelial cell line (NCM460) [21] cultured with 4.0 mg/L folate and 2680 in NCM460 with 0.4 mg/L folate. A total of 2153 genes were enrolled in this assays. The difference of 884 genes between the two cell samples was not significant. The DNA methylation levels in promoters of 712 genes were all lower in NCM460 cultured with 0.4 mg/L folate than in NCM460 with 4.0 mg/L folate and the methylation levels of 557 genes were all higher in NCM460 cultured with 0.4 mg/L folate than in NCM460 with 4.0 mg/L folate.

### Validation of MeDIP chip assay results *in vitro* and *in vivo*

#### Validation of MeDIP chip assay results in cultured cells

**Figure 1:**
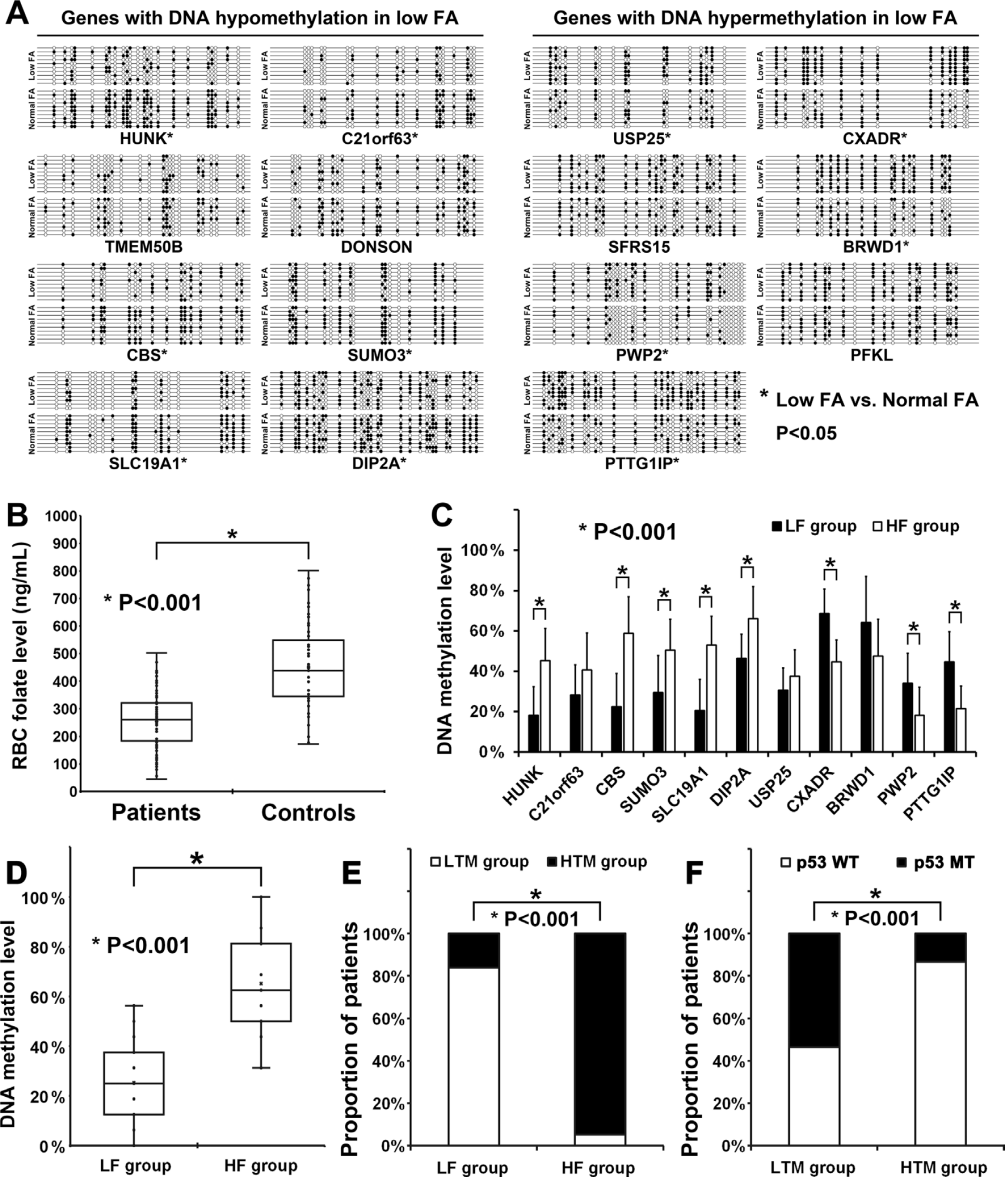
DNA hypomethylation of CBS promoter was induced by folate deficiency Folate was represented as FA. Red blood cell folate level was represented as RBC folate level. Patients with a high RBC folate level (> 276 ng/mL) and a low RBC folate level (< 276 ng/mL) were reported as HF group and LF group, respectively. Patients with a high (> 41.58%) and a low (< 41.58%) DNA methylation level of CBS promoter in tumor tissues were reported as HTM group and LTM group, respectively. (**A**) All solid and hollow ellipses represented CpG sites in CpG island of promoter. All solid ellipses represented methylated sites but all hollow ellipses represented non-methylated sites. Abnormal changes in DNA methylation levels of HUNK (low FA: 27.39% ± 9.40%, normal FA: 57.83% ± 14.35%, *P* < 0.001), C21orf63 (low FA: 17.78% ± 10.41%, normal FA: 37.78% ± 17.92%, *P* = 0.016), CBS (low FA: 30.63% ± 9.97%, normal FA: 70.63% ± 10.23%, *P* < 0.001), SUMO3 (low FA: 34.12% ± 13.53%, normal FA: 52.94% ± 14.14%, *P* = 0.011), SLC19A1 (low FA: 21.76% ± 11.78%, normal FA: 50.00% ± 7.47%, *P* < 0.001), DIP2A (low FA: 41.07% ± 7.58%, normal FA: 58.93% ± 12.74%, *P* = 0.001), USP25 (low FA: 41.18% ± 9.61%, normal FA: 26.47% ± 13.37%, *P* = 0.024), CXADR (low FA: 76.67% ± 14.14%, normal FA: 41.33% ± 13.26%, *P* < 0.001), BRWD1 (low FA: 65.29% ± 16.28%, normal FA: 37.65% ± 11.83%, *P* = 0.001), PWP2 (low FA: 32.50% ± 10.54%, normal FA: 15.00% ± 5.62%, *P* < 0.001), and PTTG1IP (low FA: 55.77% ± 15.09%, normal FA: 35.00% ± 13.98%, *P* = 0.007) were found in NCM460 cultured with low (0.4 mg/L) or normal (4.0 mg/L) FA content. No significant changes in DNA methylation levels of TMEM50B (low FA: 18.64% ± 9.45%, normal FA: 27.73% ± 13.29%, *P* = 0.127), DONSON (low FA: 29.47% ± 13.63%, normal FA: 42.11% ± 12.65%, *P* = 0.082), SFRS15 (low FA: 45.00% ± 8.69%, normal FA: 33.64% ± 15.18%, *P* = 0.067), and PFKL (low FA: 58.89% ± 17.61%, normal FA: 45.56% ± 14.30%, *P* = 0.108) were found in NCM460 cultured with low (0.4 mg/L) or normal (4.0 mg/L) FA content. (**B**) The RBC folate level of patients (256.61 ± 98.21 ng/mL) was significant lower than control people (450.87 ± 160.44 ng/mL, *P* < 0.001). (**C**) Abnormal changes in DNA methylation level of HUNK (LF group: 18.26% ± 14.02%, HF group: 45.22% ± 16.03%, *P* = 0.002), CBS (LF group: 22.50% ± 16.46%, HF group: 58.75% ± 18.21%, *P* < 0.001), SUMO3 (LF group: 29.41% ± 18.39%, HF group: 50.59% ± 15.24%, *P* = 0.019), SLC19A1 (LF group: 20.59% ± 15.50%, HF group: 52.94% ± 14.41%, *P* < 0.001), DIP2A (LF group: 46.43% ± 12.02%, HF group: 66.07% ± 15.91%, *P* = 0.011), CXADR (LF group: 68.67% ± 12.19%, HF group: 44.67% ± 10.91%, *P* < 0.001), PWP2 (LF group: 34.17% ± 14.80%, HF group: 18.33% ± 13.92%, *P* = 0.030), and PTTG1IP (LF group: 44.62% ± 14.86%, HF group: 21.54% ± 11.21%, *P* = 0.001) were found in tumor tissues of 10 patients from LF group or HF group. No significant changes in DNA methylation levels of C21orf63 (LF group: 28.33% ± 14.92%, HF group: 40.56% ± 18.34%, *P* = 0.158), USP25 (LF group: 30.59% ± 11.02%, HF group: 37.65% ± 13.07%, *P* = 0.231), and BRWD1 (LF group: 64.12% ± 22.94%, HF group: 47.65% ± 18.07%, *P* = 0.086) were found in tumor tissues of 10 patients from LF group or HF group. (**D**) The DNA methylation level of CBS promoter in tumor tissues of patients from LF group (25.22% ± 14.98%) was significant lower than patients from HF group (65.06% ± 19.22%, *P* < 0.001). (**E**) 47 of 56 patients from LF group showed a low DNA methylation level of CBS promoter in tumor tissues (LTM group) but only 2 of 39 patients from HF group showed a low DNA methylation level (LTM group).

Chromosome 21 is the smallest human chromosome and likely contains 300 to 400 genes [22]. According to the MeDIP chip assay data, a total of 15 genes (Supplementary Table 1), located in chromosome 21, showed aberrant methylation patterns (7 were higher and 8 were lower in NCM460 cultured with 0.4 mg/L folate). Therefore, above 15 genes were selected to further validate the MeDIP chip results by bisulfite sequencing PCR (BSP) in NCM460 cultured with normal (4.0 mg/L) or low (0.4 mg/L) folate content. As summarized in Figure 1A, the BSP results of 11 genes were consistent with the MeDIP chip assay results but other genes manifested no difference.

### Validation of MeDIP chip assay results in tissue samples

Firstly, the biochemical assessment for red blood cell (RBC) folate was performed with all patients and control people. As summarized in Figure 1B, the level of RBC folate in patients was significantly lower than in control people. Then, all patients were divided into high RBC folate level group (HF group, > 276 ng/mL, *n* = 39) and low RBC folate level group (LF group, < 276 ng/mL, *n* = 56). Then, tumor tissues of 10 patients from HF group and 10 from LF group were selected randomly and used to further validate above 11 genes by BSP. As summarized in Figure 1C, there were 8 genes manifested significant difference between tumor tissues of 10 patients from HF group with 10 patients from LF group.

### DNA hypomethylation of CpG islands in CBS prompter induced by folate deficiency

Above results showed that the methylation level of CpG island in promoter of cystathionine-beta-synthase (CBS), which is involved in folate metabolism and plays an important role in tumor growth and metastasis promoting [23], was significantly lower in tumor tissues of 10 patients from LF group than in tumor tissues of 10 patients from HF group (Figure 1C).

Then, the DNA methylation level of CBS promoter was further detected in tumor tissues of all 95 patients (41.58% ± 25.86%, median = 37.50%). As summarized in Figure 1D, the DNA methylation level was also significantly lower in tumor tissues of patients from LF group (25.22% ± 14.98%) than tumor tissues of patients from HF group (65.06% ± 19.22%). Then, all 95 patients were divided into high DNA methylation level group (HTM group, > mean, *n* = 46) and low methylation level group (LTM group, < mean, *n* = 49) according to the average DNA methylation level of CBS promoter in all tumor tissues (mean = 41.58%).

As summarized in Figure 1E, most patients of LF group (83.93%) showed a low methylation level of CBS promoter (LTM group), but less patients of HF group (5.13%) showed a low methylation level of CBS promoter (*P <* 0.001). In this study, the effect of low RBC folate level (LF group) and hypomethylation of CBS promoter in tumor tissues (LTM group) was very consistent in patients’ classification (Spearman = 0.776, Kappa = 0.767).

It is well-known that 5-Hydroxymethylcytosine (5-hmC) has an important role in the demethylation of DNA [24]. To verify whether DNA hypomethylation in CBS promoter was related to the demethylation of 5-hmC, the total 5-hmC level and local 5-hmC level of CBS promoter were both detected. Our results disclosed that folate deficiency decreased the total level of 5-hmC (Supplementary Figure 1) but did not have a significant effect on local 5-hmC level of CBS promoter (Supplementary Figure 2). Therefore, DNA hypomethylation in CBS promoter was mainly caused by folate deficiency but not by the demethylation of 5-hmC.

### Correlation analysis of DNA hypomethylation in CBS promoter with p53 mutation

Mutations in the tumor suppressor gene p53 are very common in CRC and p53 is an important biomarker of CRC [25]. Then, the analysis on correlation of DNA hypomethylation in CBS promoter with p53 mutations was carried out. Most tumor tissues of patients (67.37%) showed a positive p53 staining. Then, p53 mutations were further detected by DHPLC and sequencing for the 64 samples positive stained with p53. A total of 6 main types of p53 mutations existed in 33.68% tumor tissues of patients (32/95), including: p.Tyr220Cys, p.Arg196X, p.Ile232Phe, p.Arg248Gln, p.Asp228Asn. p.Arg273His, p.Gln165X, p.Arg248Leu, p.Asp259Val, p.His178Asp.

As summarized in Figure 1F, most patients of LTM group (53.06%) showed a p53 mutation and but less patients of LTM group (13.04%) showed a p53 mutation. In this study, the effect of DNA hypomethylation in CBS promoter (LTM group) and p53 mutation in tumor tissues was consistent in patients’ classification (Spearman = 0.423, Kappa = 0.396).

### Up-regulation of CBS induced by DNA hypomethylation and folate deficiency

#### Up-regulation of CBS induced by folate deficiency in cultured cells

**Figure 2:**
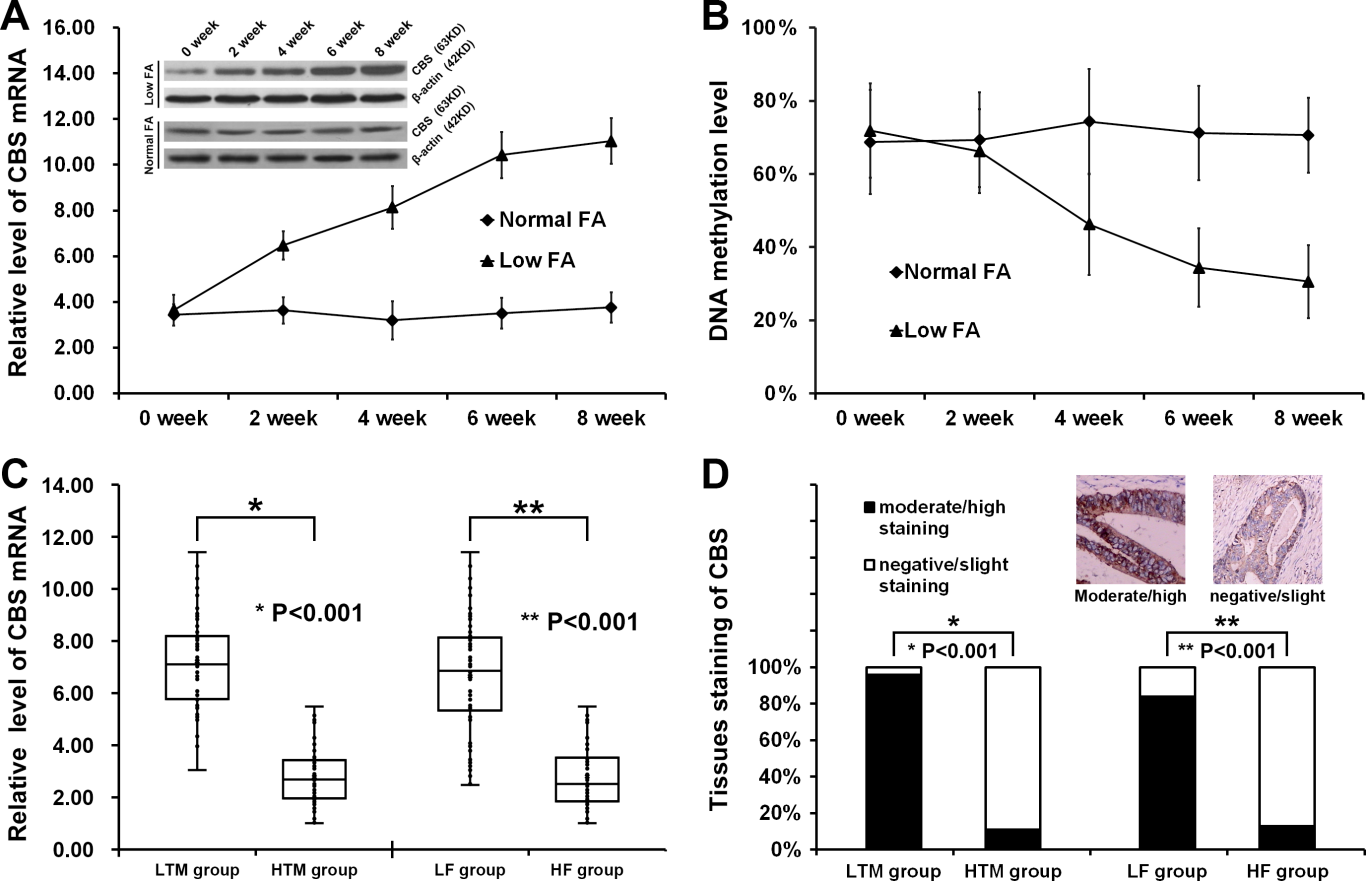
Up-regulation of CBS was induced by DNA hypomethylation and folate deficiency (**A**) The relative level of CBS mRNA in NCM460 cultured with low FA content (0.4 mg/L) gradually increased with the culture time extension (0 week: 3.64 ± 0.67, 2 weeks: 6.48 ± 0.62, 4 weeks: 8.13 ± 0.93, 6 weeks: 10.43 ± 1.02, 8 weeks: 11.04 ± 0.99, *P* < 0.001) and the level of CBS protein also gradually increased with the culture time extension. The relative level of CBS mRNA in NCM460 cultured with normal FA content (4.0mg/L) had no changes with the culture time extension (0 week: 3.44 ± 0.47, 2 weeks: 3.63 ± 0.58, 4 weeks: 3.19 ± 0.83, 6 weeks: 3.50 ± 0.67, 8 weeks: 3.76 ± 0.66, *P* = 0.765) and the level of CBS protein also had no changes with the culture time extension. (**B**) The DNA methylation level of CBS promoter cultured with low FA content (0.4 mg/L) gradually decreased with the culture time extension (0 week: 71.88% ± 12.93%, 2 weeks: 66.25% ± 11.49%, 4 weeks: 46.25% ± 13.88%, 6 weeks: 34.38% ± 10.72%, 8 weeks: 30.63% ± 9.97%, *P* < 0.001). The DNA methylation level of CBS promoter cultured with normal FA content (4.0 mg/L) had no changes with the culture time extension (0 week: 68.75% ± 14.25%, 2 weeks: 69.38% ± 12.95%, 4 weeks: 74.38% ± 14.38%, 6 weeks: 71.25% ± 12.87%, 8 weeks: 70.63% ± 10.23%, *P* = 0.671). (**C**) The relative level of CBS mRNA in tumor tissues of patients from LTM group (7.22 ± 1.87) was significant higher than tumor tissues of patients from HTM group (2.78 ± 1.12, *P* < 0.001). The relative level of CBS mRNA in tumor tissues of patients from LF group (6.65 ± 2.29) was significant higher than tumor tissues of patients from HF group (2.79 ± 1.27, *P* < 0.001). (**D**) 47 of 49 patients from LTM group showed a moderate/high CBS staining but only 5 of 46 patients from HTM group showed a moderate/high CBS staining. 47 of 56 patients from LF group showed a moderate/high CBS staining but only 5 of 39 patients from HTM group showed a moderate/high CBS staining.

Quantitative real-time PCR showed that the mRNA level of CBS was significantly higher in NCM460 cultured with 0.4 mg/L folate for 8 weeks than in cells with 4.0 mg/L folate for 8 weeks (Figure 2A). Western blotting also showed that the protein level of CBS was significantly higher in NCM460 cultured with 0.4 mg/L folate than in NCM460 with 4.0 mg/L folate (Figure 2A). With the culture time extension (0, 2, 4, 6, 8 weeks), the DNA methylation level of CBS promoter decreased gradually and the expression level of CBS increased gradually in NCM460 cultured with 0.4 mg/L folate, but the DNA methylation level of CBS promoter and the expression level of CBS were not significantly changed in NCM460 with 4.0 mg/L folate (Figure 2A and 2B).

### Up-regulation of CBS induced by hypomethylation of promoter in tissue samples

Quantitative real-time PCR showed that the mRNA level of CBS was significantly higher in tumor tissues of patients from LTM group than from HTM group (Figure 2C). As summarized in Figure 2D, most tumor tissues of patients from LTM group (95.92%) showed a moderate/high CBS staining, but less tumor tissues of patients from HTM group (10.87%) showed a moderate/high CBS staining (*P <* 0.001). In this study, the effect of hypomethylation of CBS promoter in tumor tissues (LTM group) and the moderate/high CBS staining was very consistent in patients’ classification (Spearman = 0.854, Kappa = 0.852).

### Up-regulation of CBS induced by folate deficiency in tissue samples

Quantitative real-time PCR showed that the mRNA level of CBS was higher in tumor tissues of patients from LF group than from HF group (Figure 2C). As summarized in Figure 2D, most tumor tissues of patients from LF group (83.93%) showed a moderate/high CBS staining, but less tumor tissues of patients from HF group (12.82%) showed a moderate/high CBS staining (*P <* 0.001). In this study, the effect of low RBC folate level (HF group) and the moderate/high CBS staining was very consistent in patients’ classification (Spearman = 0.703, Kappa = 0.700).

### Clinical implication of DNA methylation patterns of CBS promoter in plasma

#### Clinicopathologic features

The clinicopathologic characteristics of CRC patients are summarized in Table 1. The 95 patients (54 males, 41 females) ranged from 34 to 84 with the average age of 54.6 (10 stage I, 22 stage II, 48 stage III, and 15 stage IV) and the follow-up periods ranged from 6 to 60 months with a mean of 37.5 months. According to WHO classification standards, 36.84% (35 of 95) of cases were well-differentiated adenocarcinoma, 46.32% (44 of 95) were moderately differentiated, and 16.84% (16 of 95) were poorly differentiated. The majority (57.89%, 55 of 95) of tumors were more advanced stage (pathologic T3 stage), and 17 of 95 patients had synchronous liver metastasis at operation. 35 patients received postoperative chemotherapy or radiationtherapy. Among them, 19 patients received both chemotherapy and radiation, 12 received postoperative chemotherapy alone, and 4 received postoperative radiotherapy alone.

**Table 1:** Clinicopathologic correlation of plasma cfDNA methylation of CBS promoter

		Plasma cfDNA methylation level		
Characteristic	*n*	HPM group (*n* = 42) *n* (%)	LPM group (*n* = 53) *n* (%)	*P* value
Age	54.6 ± 11.0	37.7 ± 10.8	67.5 ± 11.4	< 0.001
Sex				
Male	54	22 (52.38%)	32 (60.38%)	0.532
Female	41	20 (47.62%)	21 (39.62%)	
Tumor location				0.806
left colon	8	5 (11.90%)	3 (05.66%)	
right colon	14	6 (14.29%)	8 (15.09%)	
sigmoid colon	16	7 (16.67%)	9 (16.98%)	
rectum	57	24 (57.14%)	33 (62.27%)	
Tumor size				0.193
≥ 5 cm	33	18 (42.86%)	15 (28.30%)	
< 5 cm	62	24 (57.14%)	38 (71.70%)	
pT stage				0.018*
pT_1_	10	8 (19.05%)	2 (03.77%)	
pT_2_	22	12 (28.57%)	10 (18.87%)	
pT_3_	55	21 (50.00%)	34 (64.15%)	
pT_4_	8	1 (02.38%)	7 (13.21%)	
pN stage				0.021*
pN_0_	45	26 (61.90%)	19 (35.85%)	
pN_1_	28	11 (26.19%)	17 (32.08%)	
pN_2_	22	5 (11.90%)	17 (32.08%)	
Liver metastasis†				0.003*
Absent	78	40 (95.24%)	38 (71.70%)	
Present	17	2 (04.76%)	15 (28.30%)	
pTNM stage				0.006*
I	10	9 (21.43%)	1 (01.89%)	
II	22	10 (23.81%)	12 (22.64%)	
III	48	20 (47.62%)	28 (52.83%)	
IV	15	3 (07.14%)	12 (22.64%)	
Dukes’ stage				0.013*
A	10	7 (16.67%)	3 (05.66%)	
B	46	25 (59.52%)	21 (39.62%)	
C	31	9 (21.43%)	22 (41.51%)	
D	8	1 (02.38%)	7 (13.21%)	
Tumor differentiation				0.095
Well	35	20 (47.62%)	15 (28.30%)	
Moderate	44	18 (42.86%)	26 (49.06%)	
Poor	16	4 (09.52%)	12 (22.64%)	
Cancer-related death				0.003*
≥ 5 years	58	33 (78.57%)	25 (47.17%)	
< 5 years	37	9 (21.43%)	28 (52.83%)	
Recurrence				0.001*
Absent	52	31 (73.81%)	21 (39.62%)	
Present	43	11 (26.19%)	32 (60.38%)	

*The clinicopathologic features which were corrected with decreased cfDNA methylation level of CBS promoter in plasma of patients.

†Synchronous liver metastasis at operation.

pTNM, pathologic tumor-node–metastasis.

### DNA methylation patterns of CBS promoter in plasma samples

**Figure 3:**
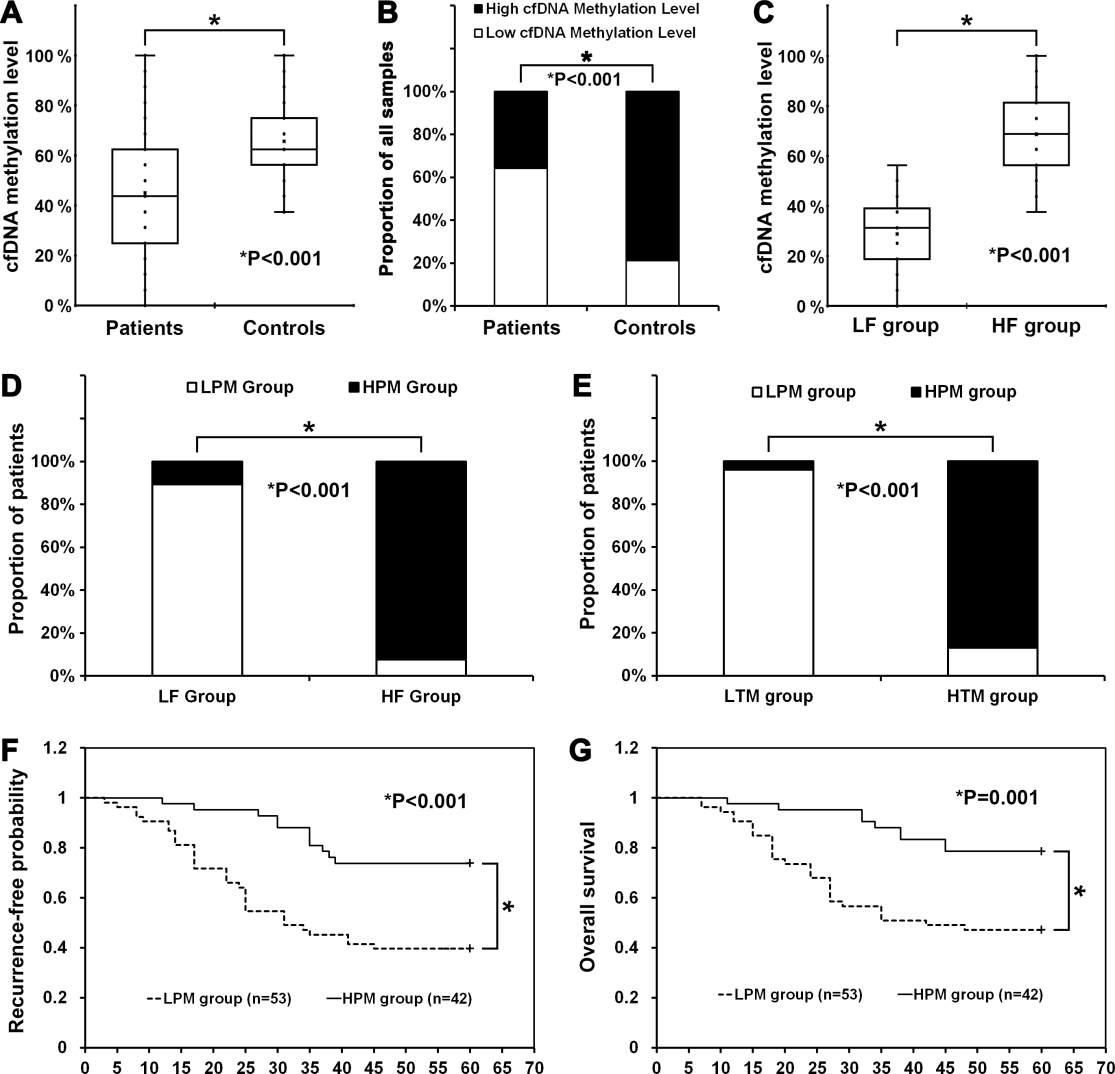
Analysis of correlation between cfDNA methylation level of CBS promoter in plasma of patients with patient characteristics and clinical outcomes Patients with a high (> 45.13%) and a low (< 45.13%) cfDNA methylation level of CBS promoter in plasma were reported as HPM group and LPM group, respectively. (**A**) The cfDNA methylation level of CBS promoter in plasma of patients (45.13% ± 24.79%) was significant lower than controls (65.82% ± 15.74%, *P* < 0.001). (**B**) Plasma samples from 95 patients and 47 controls were divided into high cfDNA methylation level and low cfDNA methylation level according to the average cfDNA methylation level of all samples (mean = 51.98%). 61 of 95 patients showed a low cfDNA methylation level of CBS promoter in plasma but 10 of 47 controls showed a low cfDNA methylation level of CBS promoter in plasma. (**C**) The cfDNA methylation level of CBS promoter in plasma of patients from LF group (28.79% ± 14.19%) was significant lower than patients from HF group (68.59% ± 16.44%, *P* < 0.001). (**D**) 50 of 56 patients from LF group showed a low cfDNA methylation level of CBS promoter in plasma (LPM group) but only 3 of 39 patients from HF group showed a low methylation level (LPM group). (**E**) 47 of 49 patients from LTM group showed a low cfDNA methylation level of CBS promoter in plasma (LPM group) but only 6 of 46 patients from HTM group showed a low methylation level (LPM group). (**F**) Patients from LPM group had increased recurrence rate compared with patients from HPM group (*P* < 0.001). (**G**) Patients from LPM group had decreased overall survival compared with patients from HPM group (*P* = 0.001).

The cfDNA of 95 patients and 47 control people were all enriched with plasma samples and used to detect the cfDNA methylation level of CBS promoter. As summarized in Figure 3A, the cfDNA methylation level of CBS promoter in plasma of patients was significantly lower than in plasma of control people. Then, all 142 plasma samples (95 patients & 47 controls) were divided into high cfDNA methylation level samples and low cfDNA methylation level samples according to the average cfDNA methylation level of all samples (mean = 51.98%). As summarized in Figure 3B, more patients (64.21%) showed a low cfDNA methylation level of CBS promoter in plasma, but less controls (21.28%) showed a low cfDNA methylation level of CBS promoter in plasma (*P* < 0.001). In our study, the specificity of low cfDNA methylation level of CBS promoter in plasma samples in diagnosis of CRC patients was high (Spearman = 0.404, Kappa = 0.380).

As summarized in Figure 3C, the cfDNA methylation level of CBS promoter in plasma of patients from LF group was also significantly lower than in plasma of patients from HF group. Finally, all 95 patients were also divided into high cfDNA methylation level group (HPM group, > mean, *n* = 42) and low cfDNA methylation level group (LPM group, < mean, *n* = 53) according to the average cfDNA methylation level of all patients (mean = 45.13%).

As summarized in Figure 3D, most patients from LF group (89.29%) showed a low cfDNA methylation level of CBS promoter in plasma (LPM group), but less patients from HF group (7.69%) showed a low cfDNA methylation level (*P* < 0.001). In this study, the effect of low RBC folate level (LF group) and low cfDNA hypomethylation level of CBS promoter in patient plasma (LPM group) was very consistent in patients’ classification (Spearman = 0.808, Kappa = 0.807).

As summarized in Figure 3E, most patients of LTM group (95.92%) showed a low cfDNA methylation level of CBS promoter in plasma (LPM group), but less patients of HTM group (13.04%) showed a low cfDNA methylation level (*P* < 0.001). The effect of hypomethylation of CBS promoter in cfDNA of patient plasma (LPM group) and in DNA of tumor tissues (LTM group) was very consistent in patients’ classification (Spearman = 0.834, Kappa = 0.831).

### Clinicopathologic correlation

As summarized in Table 1, low cfDNA methylation level of CBS promoter in plasma of patients (LPM group) was associated with age (*P <* 0.001), pT stage (*P* = 0.018), pN stage (*P* = 0.021), liver metastasis (*P* = 0.003), pTNM stage (*P* = 0.006), advanced Dukes’ stage (*P* = 0.013), recurrence (*P* = 0.001), and five-year survival (*P* = 0.003). Take together the incidence of recurrence was significantly higher in patients of LPM group (60.38%) than HPM group (26.19%), and 5-year survival rate was significantly lower in patients of LPM group (52.83%) than HPM group (21.43%). On the other hand, no significant differences were observed regarding sex (*P* = 0.532), tumor location (*P* = 0.806), tumor size (*P* = 0.193), and tumor differentiation (*P* = 0.095).

### Univariate and multivariate analyses of recurrence and survival

The median follow-up time was 19 months and the mean follow-up time was 37.5 months. In Univariate analysis (Table 2), there is increased risk of recurrence with increasing age (≥ 55 vs. < 55, *P* = 0.021), pT stage (T3 vs. T2, *P* = 0.026; T4 vs. T3, *P* = 0.023), pN stage (N2 vs. N1, *P* = 0.009), liver metastasis (*P* = 0.004), pathologic TNM stage (II vs. I, *P* = 0.042; III vs. II, *P* = 0.029; IV vs. III, *P* = 0.006), and cfDNA methylation level of CBS promoter in plasma of patients (LPM group vs. HPM group, *P* = 0.005). There is also increased risk of cancer-related death with increasing age (≥ 55 vs. < 55, *P* = 0.035), pT stage (T3 vs. T2, *P* = 0.030, T4 vs. T3, *P* = 0.035), pN stage (N2 vs. N1, *P* = 0.023), liver metastasis (*P* = 0.004), pathologic TNM stage (III vs. II, *P* = 0.033; IV vs. III, *P* = 0.009), and cfDNA methylation level of CBS promoter in plasma of patients (LPM group vs. HPM group, *P* = 0.009). Kaplan-Meier curves obtained with two categories resulted in statistically significant correlation of patients of LPM group with decreased tumor recurrence-free probability (Figure 3F) and overall survival (Figure 3G).

**Table 2:** Univariate analysis of recurrence-free and overall survivals

		Overall survival		Recurrence-free probability	
Characteristic	*n*	RR (95% CI)	*P* value	RR (95% CI)	*P* value
Age					
< 55 years	41	1.00		1.00	
≥ 55 years	54	1.43 (1.18–3.56)	0.035	1.61 (1.26–4.79)	0.021
Sex					
Female	54	1.00		1.00	
Male	41	1.21 (0.77–1.92)	0.438	1.28 (0.85–2.01)	0.337
pT stage			0.034*		0.022*
pT_1_	10	1.00		1.00	
pT_2_	22	4.69 (0.61–38.51)	0.229	6.91 (0.88–49.95)	0.165
pT_3_	55	8.83 (1.25–61.95)	0.030	9.73 (1.28–69.26)	0.026
pT_4_	8	9.27 (1.21–70.33)	0.035	10.14 (1.37–81.42)	0.023
pN stage			0.018†		0.009†
pN_0_	45	1.00		1.00	
pN_1_	28	5.15 (0.76–41.13)	0.102	2.83 (0.98–9.37)	0.071
pN_2_	22	10.91 (1.56–77.48)	0.023	7.17 (1.79–20.15)	0.009
Liver metastasis					
Present	78	1.00		1.00	
Absent	17	2.14 (1.34–4.95)	0.004	2.15 (1.41–5.07)	0.004
pTNM stage			0.009‡		0.005‡
I	10	1.00		1.00	
II	22	3.16 (0.73–13.79)	0.105	8.15 (1.12–59.96)	0.042
III	48	4.94 (1.21–19.82)	0.033	8.99 (1.21–65.75)	0.029
IV	15	8.79 (2.02–38.78)	0.009	18.53 (1.95–137.72)	0.006
CBS methylation					
HPM group	42	1.00		1.00	
LPM group	53	1.49 (1.15–2.64)	0.009	1.62 (1.29–3.68)	0.005

*Overall *P* value for pT stage.

†Overall *P* value for pN stage.

‡Overall *P* value for pTNM stage.

95% CI, 95% confidence interval; pTNM, pathologic tumor-node–metastasis; RR, risk ratio.

Multivariate analysis indicated that low cfDNA methylation level of CBS promoter in patient plasma (LPM group) and liver metastasis remained as significant independent prognostic factors of increased tumor recurrence rate (*P* = 0.025 and *P* = 0.017, respectively) and overall survival (*P* = 0.027 and *P* = 0.021, respectively) for patients (Table 3).

**Table 3:** Multivariate Cox regression analysis of recurrence and overall survivals

		Overall survival		Recurrence-free probability	
Characteristic	*n*	RR (95% CI)	*P* value	RR (95% CI)	*P* value
pT stage			0.164*		0.128*
pT_1_	10	1.00		1.00	
pT_2_	22	3.62 (0.51–13.19)	0.349	4.17 (0.58–16.95)	0.274
pT_3_	55	5.76 (0.94–16.12)	0.097	6.16 (0.91–19.92)	0.090
pT_4_	8	5.15 (0.77–27.71)	0.105	8.73 (0.84–20.87)	0.075
pN stage			0.101†		0.095†
pN_0_	45	1.00		1.00	
pN_1_	28	1.97 (0.75–8.19)	0.184	2.26 (0.85–6.37)	0.159
pN_2_	22	4.18 (0.89–14.42)	0.077	5.82 (0.98–16.75)	0.057
Liver metastasis					
Present	78	1.00		1.00	
Absent	17	1.91 (1.18–3.26)	0.025	2.15 (1.28–4.91)	0.017
CBS methylation					
HPM group	42	1.00		1.00	
LPM group	53	1.35 (1.09–2.41)	0.027	1.54 (1.18–3.02)	0.021

*Overall *P* value for pT stage.

†Overall *P* value for pN stage.

95% CI, 95% confidence interval; RR, risk ratio.

## DISCUSSION

It is generally known that epigenetic processes, including DNA methylation, are important in development and may be misdirected and predispose to tumorigenesis [5–7]. However, exact DNA methylation alterations in tumorigenesis are still unclear. In our study, aberrant DNA methylation patterns overlapping the promoter region of the relevant transcript (–800 bp ~ +200 bp) were screened by NimbleGen MeDIP chip assay in normal human colon mucosal epithelial cell line (NCM460), which was cultured with a normal or low folate content. Some data of high-throughput screening was further validated by BSP both *in vitro* and *in vivo*, and then our attention was focused on CBS, one important regulatory gene of folate metabolism and tumorigenesis [26]. The detection and correlation analysis of RBC folate level with CBS DNA methylation level were carried out for all patients and control people enrolled in our study. In addition, the detection on CBS expression and the correlation analysis of CBS expression with RBC folate level or CBS DNA methylation level were also carried out for all patients and control people. Our study showed that the long-term folate deficiency could lead to the DNA hypomethylation of CpG islands in CBS promoter, which would then result in up-regulation of CBS. Although there have been studies on genetic or epigenetic regulation of CBS and its role in tumorigenesis [27–28], to the best of our knowledge there are seldom reported studies on the DNA hypomethylation of promoter and consequent up-regulation induced by folate deficiency in CRC.

Previous studies have proved that 5-Hydroxy- methylcytosine (5-hmC) have an important role in the demethylation of DNA [24]. However, despite folate deficiency can lead to reduce the total level of 5-hmC, our results disclosed that DNA hypomethylation in CBS promoter was mainly caused by folate deficiency but not by the demethylation of 5-hmC. Therefore, further studies on mechanisms of folate deficiency leading to the decrease of total 5-hmC level and DNA hypomethylation in the absence of significant changes of local 5-hmC levels would help us to understand the role of DNA methylation in tumorigenesis.

It is well-known that mutations in the tumor suppressor gene p53 are very common in CRC and p53 is an important biomarker of CRC [25]. In our study, DNA hypomethylation of CBS promoter was positively correlated with p53 mutations. Recent findings revealed novel stress response mechanisms to 5-FU, which function independently of p53 and lead to mitochondrial apoptosis through a molecular mechanism involving CBS [29]. Therefore, as an important enzyme involved in folate metabolism [23], the interaction between CBS and p53 may play an important role in tumorigenesis and need more in-depth discussion.

CRC is most common cancer in the world and the morbidity and mortality increased quickly in China [1, 30]. Despite curative therapy, most patients still have a disease relapse leading to morbidity and eventual mortality in 5 years [1, 2]. The most important prognostic indicators for CRC continue to be tumor stage and prognostic or predictive markers [31]. However, useful prognostic factors that relate to tumor stage and clinical outcome in these patients are poorly defined. In recent years the search for noninvasive biomarkers for CRC has become a rapidly growing area of clinical research and the discovery of circulating cell-free DNA in the peripheral blood has created a new approach [32, 33]. Circulating cfDNA in plasma is quite stable and characteristic of tumor tissues [32, 34]. A large number of new researches sufficiently attested that as a biomarker circulating cfDNA methylation is very competitive [33, 35]. In our study, the methylation level of CBS promoter in plasma cfDNA of patients and controls were also detected and analyzed. Our results indicated a cfDNA hypomethylation of CBS promoter in patients and the methylation variation of CBS promoter in plasma cfDNA is strikingly consistent with tumor tissues. Even more important, patients with cfDNA hypomethylation of CBS promoter in plasma were correlated well with high tumor stage and poor clinical outcome of CRC. Statistical analysis showed that DNA hypomethylation of CBS promoter in plasma cfDNA could serve as a potential noninvasive circulating biomarker. Several cfDNA molecules had been identified as potential prognostic indicators for CRC [32, 36]; however, few methylation variations of cfDNA were identified as biomarkers for CRC. In our opinion, our new findings may be helpful in developing more effective diagnosis and prognostic markers for CRC.

As a key vitamin, the intake of folate has gotten more and more attention and an inverse relationship between folate intake and risk of CRC has already been demonstrated in follow-Up studies [3, 17, 37]. Our study showed the association between folate deficiency and tumorigenesis of CRC once again. Because of the role of folate as methyl donor and action in methyl group metabolism, the most important potential mechanism for carcinogenesis mediated by folate deficiency is aberrant global hypometylation or regional hypermethylation [16, 18, 23]. And CBS is one key regulatory enzyme in folate metabolism and plays an important role in promoting cellular bioenergetics, proliferation, and migration of cancer cells. [26–28, 38]. Therefore, the finding, that folate deficiency would induce DNA hypomethylation of CBS, in our study was reasonable and particularly important in understanding the role of folate in tumorigenesis of CRC. In addition, another outcome in our study that interested us was that, even in folate deficiency, the promoter CpG islands of some genes, such as CXADR, PWP2 and PTTG1IP, still presented a state of hypermethylation. We believe that further investigation on these genes may be helpful in revealing mechanisms of folate.

In a word, long-term folate deficiency induces DNA hypomethylation of CBS promoter in CRC and then results in up-regulation of CBS. The cfDNA hypomethylation of CBS promoter in plasma cfDNA of patients is a potential noninvasive circulating biomarker for CRC. Our study may be helpful in developing more effective early diagnosis and prognostic markers for CRC.

## MATERIALS AND METHODS

### Cell culture

The normal human colon mucosal epithelial cell line (NCM460) [21] used in our studies was obtained from INCELL (San Antonio, TX, USA) and maintained in DMEM (with 1000mg/L glucose and without L-glutamine, sodium bicarbonate, folate) medium (D2429, Sigma-Aldrich, MO, USA) supplemented with 15% (v/v) KnockOut^TM^ Serum Replacement (10828–028, Thermo Fisher Scientific, CA, USA), and Penicillin-Streptomycin (100×) (15140–122, Thermo Fisher Scientific, CA, USA). NCM460 was maintained for 8 weeks in medium containing normal (4.0 mg/L) or low (0.4 mg/L) concentrations of folate according to the instructions of the medium.

### Case collection

From March 2007 to March 2010, a total of 95 consecutive patients (54 males and 41 females) with sporadic CRC, undergone surgical treatment without preoperative chemotherapy or radiotherapy, were enrolled in our studies (Table 1). Furthermore, a total of 47 control people with endoscopically normal colon in the Healthcare Center of Hangzhou Sanatorium was also enrolled. Written informed consent was obtained prior to the study. The research protocol and consent form were approved by the Ethics Review Committee of Second Military Medical University.

### NimbleGen methylated DNA Immunoprecipitation (MeDIP) chip array

The MeDIP chip array (NimbleGen, WI, USA), which cover most of the CpG islands in the human genome, was used to comprehensively analyze methylation patterns of the cell samples cultured with normal or low concentrations of folate. Chip assay and data analysis were entrusted to the KangChen Bio-tech (Shanghai, China). Briefly, the peaks data contains all identified peaks overlapping CpG islands was collected. Then the peaks data contains all peaks overlapping the promoter region of the relevant transcript (–800 bp ~ +200 bp) was analyzed and the report listing only the nearest peak to a transcript was offered.

### Tissue, whole blood, and plasma samples preparation

All tumor tissues were taken from vital areas of adenocarcinomas confirmed by three experienced pathologists and those in which tumor cells occupied a major component (> 80%) were chosen, 4–5 cm distal to the resection margin from the same resected adenocarcinoma respectively were used as adjacent control tissues. Tissues were frozen in liquid nitrogen immediately after surgical resection and kept at –80°C until DNA/RNA extraction and immunoassay.

Whole blood samples were collected from 95 patients prior to treatment or 47 control people into tubes containing EDTA (BD Biosciences, MD, USA) and partly processed for plasma isolation within 2 hour of collection. To obtain plasma, the whole blood samples were centrifuged as follows, after the first centrifugation at 1600 × g for 10 min, plasma supernatants were carefully transferred to a new tube and centrifuged again at 16,000 × g for 10 min. All Whole blood samples and plasma samples were immediately stored at –80°C until DNA extraction or further assay.

### 5-hmC detection

Dot blot assays now were performed as previously described [39] to quantify total 5-hmC level in NCM460. In brief, genomic DNA was prepared in 2×SSC buffer and denatured for 10 min at 95°C. Samples were rapidly chilled for 5 min and then spotted on a positive charged nylon membrane (Roche Diagnostics, Mannheim, Germany). The membrane was washed twice in 2×SSC buffer, UV crosslink and dried for 1 hour at 70°C, and then blocked with 5% casein buffer and incubated with anti-5-hmC antibody (Active Motif, Cat. 39769, dilution at 1:10000) at 4°C overnight. After incubation with species-specific HRP-conjugated secondary antibody (ZSGB-BIO, Cat. ZB-23012, dilution at 1:5000), dot signal was visualized with the Western Bright ECL detection system (Advansta, CA) by exposing to X-ray film. To ensure equal spotting of total DNA on the membrane, the same blot was stained with 0.02% methylene blue in 0.3 M sodium acetate (pH 5.2). The 5-hmC intensity was finally quantified by image processing and analysis.

The local level of 5-hmC in CBS promoter was detected by Tet-assisted sodium bisulfite sequencing (TAB-seq) in NCM460. The 5-hmC TAB-Seq Kit (A-K0010, A&D Technology, Beijing, China) was used. Genomic DNA was extracted as described above. One microgram of sonicated genomic DNA was treated with the 5-hmC TAB-Seq Kit according to the instructions. The resultant PCR products were size-fractionated on a 1.0% agarose gel, purified, and cloned into pMD^TM^19-T Vector (6013, TaKaRa). Finally, ten clones were sequenced to ensure that we would have the power to calculate statistical significance of any findings.

### p53 mutation detection

p53 status in tumor samples was determined by immunohistochemistry staining. In brief, antigen retrieval was carried out on formalin-fixed paraffin-embedded tissue sections for 20 minutes at 95°C using Target retrieval solution (pH 6.0; Dako-Cytomation, Carpinteria, CA). Immunolabeling was performed with a mouse monoclonal antibody (clone DO-7; Dako Cytomation) at 2.5 μg/ml. Immunocomplexes were labeled with a biotinylated anti-mouse secondary antibody, an avidin–biotin–horseradish peroxidase complex (Vector Laboratories, Burlingame, CA), and the 3,3′-diaminobenzidine chromogen. Nuclear immunostainning for p53 was calculated as the percentage of positive epithelial cells in relation to the total number of cells encountered in at least 5–10 representative high power fields (500–1000 epithelial cells). The immunoreactivity was interpreted by means of light microscopic examination and evaluated independently by three experienced pathologists. The staining was evaluated only in the areas with well-preserved tissue morphology and away from necrosis or artefacts. Tumors were scored as being positive for over-expression if nuclear staining was evident in at least 10% of neoplastic cells, consistent with other published analyses [40].

For the samples positive stained with p53, we further detected p53 mutation by DHPLC and sequencing method. Briefly, Genomic DNA was extracted from 10 mg of each of the tumor samples, using the Dneasy tissue Kit column (Qiagen, Valencia, CA, USA). Exons 2–9 of p53 was PCR amplified. PCR products were then performed dHPLC analysis, fragments that displayed an abnormal chromatogram at any temperature were bi-directionally sequenced using an ABI 3100 Genetic Analyzer (Applied Biosystems, Foster City, CA, USA).

### Biochemical assessment for red blood cell (RBC) folate

To reflect the long-term folic acid stores of patients [41], the levels of RBC folate were measured by an electrochemical luminescence (ECL) immunoassay on Beckman Coulter Access^®^ 2 immunoassay analyzer (Beckman Coulter, CA, USA) with the correlative kit for RBC folate applications (Beckman Coulter). Immunoassay and data analysis were entrusted to the Intertek Medical Testing Center (MDTC) (Shanghai, China). The expected normal values (ENV) for RBC folate were more than 276 ng/ml (625 nmol/L) packed RBC according to the manufacturer's instructions. Folate deficiency was defined when a RBC folate level was less than 276 ng/ml.

### DNA isolation

DNA isolation from cell samples (5 × 10^6^/case) or tissue samples (25 mg/case) was performed with DNeasy Blood & Tissue Kit (50) (69504, QIAGEN, Hilden, Germany) according to the instructions. cfDNA isolation from plasma samples (5 ml/case) was performed with QIAamp Circulating Nucleic Acid Kit (50) (55114, QIAGEN) according to the instructions. DNA was dissolved in 80 μl elution buffer (EB) and stored at –80°C until use in subsequent experiments. The quality control for purified DNA was performed by detecting A_260_/A_280_ ratio.

### Bisulfite conversion of DNA and bisulfite sequencing PCR (BSP)

Sodium bisulfite conversion and DNA recovery were performed with EpiTect Bisulfite Kit (48) (59104, QIAGEN, Germany) according to the manufacturer's instructions. Bisulfite-converted DNA was then re-suspended in 40 μl EB and stored at –80°C until use in subsequent experiments.

A total of 10ng bisulfite-converted DNA was amplified by PCR and all primers are listed in Supplementary Table 2. All PCR reactions were performed on Applied Biosystems^®^ Veriti^®^ Thermal Cycler (Thermo Fisher Scientific, CA, USA) in a total volume of 25μl using TaKaRa LA Taq^®^ (RR02MA, TaKaRa, Japan). Then, PCR products were cloned into the pMD^TM^19-T Vector and ten clones were sequenced using Applied Biosystems^®^ 3130 Genetic Analyzer (Thermo Fisher Scientific). Then the sequencing results were analyzed and determined the methylation ratio (the number of methylated CpG sites/the number of total CpG sites) of CpG island in each sequencing clone. The mean of 10 clones of each sample was used as the methylation ratio of the sample.

### RNA isolation, reverse transcription (RT) reaction and quantitative real-time PCR

Total RNA was purified with a Trizol^®^ Reagent kit (Thermo Fisher Scientific) and treated with DNase I to remove trace amounts of DNA contamination. RT reaction was performed with an M-MLV Reverse Transcriptase kit (Thermo Fisher Scientific). Quantitative real-time PCR was performed by SYBR Green detection with StepOne^TM^ Plus Real-Time PCR System (Thermo Fisher Scientific). The relative level of CBS mRNA was normalized using the 2^-ΔΔCT^ method relative to the β-actin internal reference. Data were analyzed by the StepOne^TM^ Software (version 2.1, Thermo Fisher Scientific). All primers are listed in Supplementary Table 2.

### Western blot

Western blot was performed with monoclonal anti-CBS antibody (sc-100519, Santa Cruz, USA), horseradish peroxidase (HRP)-conjugated secondary antibodies (R&D, MN, USA) and substrate. Briefly, the harvested cells were lysed in 10 mM Tris-HCl (pH 7.6), 1 mM EDTA, 400 mM NaCl, 10% glycerol, 5 mM β-mercaptoethanol and a protease inhibitor cocktail. Lysates were incubated on ice for 10 min and then centrifuged at 8,000 × g to remove cellular debris. Forty to fifty micrograms of protein were used for Western transfer and immunobloting. Ponceau S staining of membranes verified proper transfer of proteins to nitrocellulose membranes. Monoclonal anti-CBS antibody were used. A HRP-conjugated secondary antibody was incubated for 2 hours at room temperature, and the corresponding band was revealed using the DAB method (Sigma, MO, USA). As control for equal loading of the samples, the membranes were re-probed with β-actin.

### Tissue immunohistochemistry

Tissues were identified on H&E-stained slides, and then immunohistochemistry was performed with monoclonal anti-CBS antibody. Briefly, Slides were fixed with methanol–acetone (1:1) at −20°C for at least 10min and rehydrated in PBS for 15 min at room temperature. Monoclonal anti-CBS antibody was diluted in blocking buffer (2% BSA, 0.2% Tween-20, 10% glycerol in PBS) and incubated with the slides for 60min at room temperature followed by three washes in PBS. The HRP-conjugated secondary antibodies were diluted in blocking buffer and incubated with the slides for an additional 45 min. To detect nuclei, hematoxylin (Sigma) was added. After three washes in PBS, DAB substrate (Sigma) was added for 20 min and the reaction was then terminated. At last, the slides were dehydrated with alcohol and mounted with mountant solutions. Images were collected using a confocal scanning microscope TCS SP2 (Leica Microsystems).

The protein levels were graded comprehensively by the staining range and extent based on a modified Fromowitz standard [42]. Briefly, 5 random visual fields were observed and 100 cells were counted in each field. The average ratio of stained cells in 5 fields was regarded as the positive range score. Positive range scores were graded as follows: 0, 0–5%; l, 6–25%; 2, 26–50%; 3, 51–75%; 4, >75%. Positive extent scores were graded as follows: 0, no staining; 1, light yellow; 2, brown; 3, dark brown. Combining above two scores generated two options: negative/slight staining (< 4) and moderate/high staining (≥ 4).

### Statistical analysis

The data is updated using the latest information on the vital status provided by telephone follow-up or every time the patient comes for a follow-up visit. Overall survival was calculated from time of surgery to time of death from any cause or to time of last follow-up. Cumulative recurrence-free probability was calculated using the time between surgery date and first recurrence date (if recurred) or last follow-up date (if did not recur).

Data are presented as mean ± standard deviation (SD). The Mann-Whitney *U* test was used for two independent samples. The Wilcoxon test was used for paired samples. The Fisher's exact test was used for categorical data. The Kaplan-Meier method was used in survival and recurrence-free analysis and the log-rank test was used to compare the statistical significance. The univariate Cox regression analysis was used to determine the significance of clinical and pathologic characteristics. Cox proportional hazards models were fitted for multivariate analysis. Statistical analyses were performed using the SPSS (version 22.0, IBM, NY, USA). A two-sided significance level of 0.05 was used for all statistical analyses.

## SUPPLEMENTARY MATERIALS FIGURES AND TABLES





## Abbreviations

5-hmC: 5-hydroxymethylcytosine
BSP: Bisulfite sequencing PCR
cfDNA: Cell-free DNA
CRC: Colorectal cancer
CBS: Cystathionine-beta-synthase
EB: Elution buffer
HF group: High RBC folate level group
HTM group: High tissue DNA methylation level group
HPM group: High cfDNA methylation level group
LF group: Low RBC folate level group
LTM group: Low tissue methylation level group
LPM group: Low cfDNA methylation level group
MeDIP: Methylated DNA Immunoprecipitation
RBC folate: Red blood cell folate
TAB-seq: Tet-assisted sodium bisulfite sequencing
