# Supplementary material for: DNA hypomethylation of CBS promoter induced by folate deficiency is a potential noninvasive circulating biomarker for colorectal adenocarcinomas
Source: Oncotarget. 2017 May 18;8(31):51387–401. doi: 10.18632/oncotarget.17988 (PMC5584256; doi:10.18632/oncotarget.17988)
Supplement: Supplementary file 1 [file oncotarget-08-51387-s001.pdf]

DNA hypomethylation of CBS promoter induced by folate deficiency is a potential noninvasive circulating biomarker for colorectal adenocarcinomas

Supplementary Materials

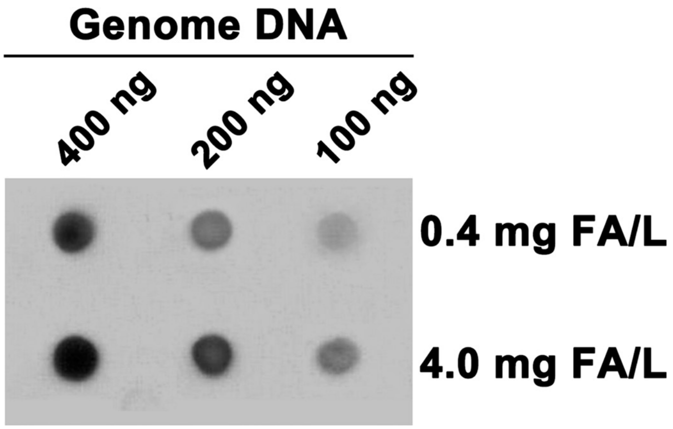

Supplementary Figure 1: The total 5-hmC level in NCM460 cultured with normal or low folate content. The gray value of the hybridization spots was positively correlated with the total level of 5-hmC. Under three different loading quantity of genome DNA (100 ng, 200 ng, 400 ng), The gray values of the hybridization spots of NCM460 cultured with normal folate content (4.0 mg/L) were all greater than the hybridization spots of NCM460 cultured with low folate content (0.4 mg/L).

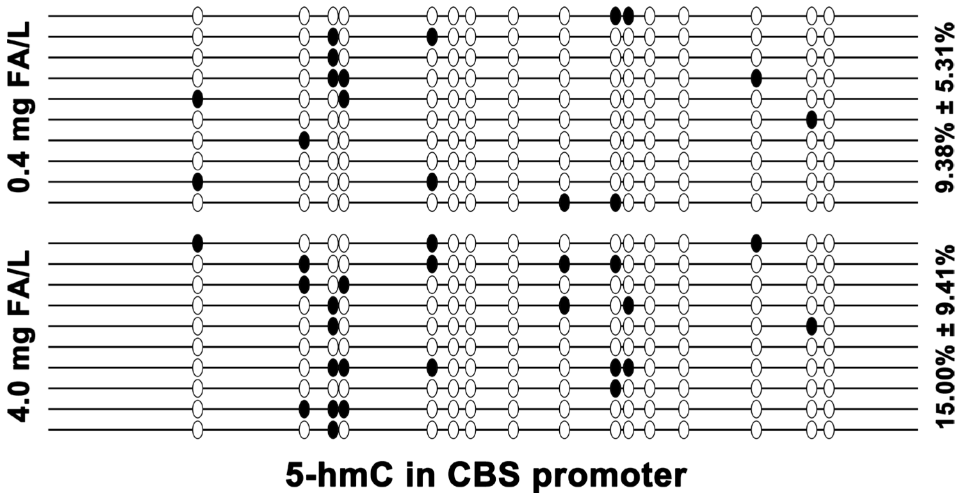

Supplementary Figure 2: The local 5-hmC level of CBS promoter in NCM460 cultured with normal or low folate content. All solid and hollow ellipses represented CpG sites in CpG island of promoter. All solid ellipses represented 5-hmC sites but all hollow ellipses represented non-5-hmC sites. In NCM460 cultured with normal folate content (4.0 mg/L), the average local 5-hmC level of CBS promoter was 15.00% ± 9.41%. In NCM460 cultured with low folate content (0.4 mg/L), the average local 5-hmC level of CBS promoter was 9.38% ± 5.31%. There was no significant difference in local 5-hmC level between NCM460 cultured with normal with low folate content (P = 0.152).

Supplementary Table 1: The fifteen genes with aberrant methylation patterns located in chromosome 21. See Supplementary_Table_1

Supplementary Table 2: Primers sequences enrolled in this study

	Name	Sequence	Product
Primers for clone of CpG islands of 15 genes located in chromosome 21	USP25-F	5′ AGGGTTATAATGTAGGGTTAGGG 3′	178 bp
	USP25-R	5′ CTACTTTAAAAAAAAAAAAAAAAAAAAC 3′	
	CXADR-F	5′ GGGAGTTTGGGATTAGGAG 3′	188 bp
	CXADR-R	5′ CCCCATTATTCCTAAATAACC 3′	
	SFRS15-F	5′ TAATTAGGAGGTAAGGGGTG 3′	225 bp
	SFRS15-R	5′ AAATAAACAATAAAAAAAAAAAAATAC 3′	
	BRWD1-F	5′ TTGTTGGTTTGTGGGGGAT 3′	164 bp
	BRWD1-R	5′ CAAAAAAACTTTTAAAATAAAAATTAACTC 3′	
	PWP2-F	5′ GGATTTTATTTATAATTTTTTATTTAATAG 3′	272 bp
	PWP2-R	5′ CCCAAAAAAACAAAAAAAAACTAC 3′	
	PFKL-F	5′ TTGGTTTTTTTTGGTTTTGAAG 3′	173 bp
	PFKL-R	5′ TCCCCAACTAAACCCACAAA 3′	
	PTTG1IP-F	5′ TGTTTTTGTTTTTGTTTTGTTTAG 3′	240 bp
	PTTG1IP-R	5′ CCATCACCCTACAAAAACCTA 3′	
	HUNK-F	5′ TTTTAAGAGGAAGAATTTTTGGG 3′	203 bp
	HUNK-R	5′ AACTCCTCCTCCCCAAACTC 3′	
	C21orf63-F	5′ GGTTTAGAAAGATGTTGTGGTTTA 3′	245 bp
	C21orf63-R	5′ TCCACAAAAAAAAAAAACTAAACC 3′	
	TMEM50B-F	5′ GAGTATTTTTTTTATTTTGGGTAGTT 3′	269 bp
	TMEM50B-R	5′ AAATAAACTACTCTACAAACCTCCC 3′	
	DONSON -F	5′ AGTTAGTTGTGAATGGGGGAT 3′	207 bp
	DONSON -R	5′ TAAAACCCCCTCACTTTCCT 3′	
	CBS-F	5′ GAAGGGTAAGAAGTTAATTAAGTAAAATA 3′	202 bp
	CBS-R	5′ ACATACCCCCACCCCAATC 3′	
	SUMO3-F	5′ GTTTTTTTTTTAGTTGGGAAGG 3′	161 bp
	SUMO3-R	5′ CAACCAACAATAACTCAAAAAAA 3′	
	SLC19A1-F	5′ TAGGGTTTTGTGAGGTGAGTG 3′	226 bp
	SLC19A1-R	5′ CCAAACCCCAAACTACAAAC 3′	
	DIP2A-F	5′ AAAGGAGTGAATATAGGTAAAGGT 3′	239 bp
	DIP2A-R	5′ CCTAAATAAAAATAACCAACCC 3′	
Sequencing	Primer RV-M	5′ GAGCGGATAACAATTTCACACAGG 3′	
	Primer M13-47	5′ CGCCAGGGTTTTCCCAGTCACGAC 3′	
Primer for quantitative of CBS	CBS-Real-F	5′ CCGACTCAGTGCGGAACTACAT 3′	266 bp
	CBS-Real-R	5′ GTTCCCAAGCGTCACCATTCC 3′	
	β-actin-Real-F	5′ GGGAAATCGTGCGTGACATTAAG 3′	275 bp
	β-actin-Real-R	5′ TGTGTTGGCGTACAGGTCTTTG 3′	
