# Supplementary material for: DNA hypomethylation of CBS promoter induced by folate deficiency is a potential noninvasive circulating biomarker for colorectal adenocarcinomas
Source: Oncotarget. 2017 May 18;8(31):51387–401. doi: 10.18632/oncotarget.17988 (PMC5584256; doi:10.18632/oncotarget.17988)
Supplement: Supplementary file 2 [file oncotarget-08-51387-s002.doc]

| Gene  Name | S_  Low | S_  Normal | Positive  Sample  Count | Accession | Chromosome | Strand | Trans  Start | Trans  End | Locus  Link  ID | Protein  ID | Protein  Name | CpG  Name | CpGS_  Low | CpGS_  Normal | CpG  Positive  Sample  Count | Relative  Location |
| --- | --- | --- | --- | --- | --- | --- | --- | --- | --- | --- | --- | --- | --- | --- | --- | --- |
| USP25 | 1 |  | 1 | NM_013396 | chr21 | + | 16024366 | 16174248 | 29761 | NP_037528 | ubiquitin specific protease 25 | chr21:16023724-16024871 | 1 |  | 1 | Promoter |
| CXADR | 1 |  | 1 | NM_001338 | chr21 | + | 17807200 | 17861137 | 1525 | NP_001329 | coxsackie virus and adenovirus receptor | chr21:17806677-17807982 | 1 |  | 1 | Promoter |
| SFRS15 | 1 |  | 1 | NM_020706 | chr21 | - | 32026302 | 31965183 | 57466 | NP_065757 | splicing factor, arginine/serine-rich 15 isoform | chr21:32025327-32026846 | 1 |  | 1 | Promoter |
| HUNK |  | 1 | 1 | NM_014586 | chr21 | + | 32167498 | 32298248 | 30811 | NP_055401 | hormonally upregulated Neu-associated kinase | chr21:32166781-32168480 |  | 1 | 1 | Promoter |
| C21orf63 |  | 1 | 1 | NM_058187 | chr21 | + | 32706622 | 32809568 | 59271 | NP_478067 | hypothetical protein LOC59271 | chr21:32705785-32707588 |  | 1 | 1 | Promoter |
| TMEM50B |  | 1 | 1 | NM_006134 | chr21 | - | 33774151 | 33743317 | 757 | NP_006125 | transmembrane protein 50B | chr21:33773099-33774572 |  | 1 | 1 | Promoter |
| DONSON |  | 1 | 1 | NM_017613 | chr21 | - | 33882884 | 33872080 | 29980 | NP_060083 | downstream neighbor of SON | chr21:33882258-33883122 |  | 1 | 1 | Promoter |
| BRWD1 | 1 |  | 1 | NM_018963 | chr21 | - | 39607426 | 39479273 | 54014 | NP_061836 | bromodomain and WD repeat domain containing 1 | chr21:39606240-39608194 | 1 |  | 1 | Promoter |
| CBS |  | 1 | 1 | NM_000071 | chr21 | - | 43369109 | 43346371 | 875 | NP_000062 | cystathionine-beta-synthase | chr21:43367693-43370058 |  | 1 | 1 | Promoter |
| PWP2 | 1 |  | 1 | NM_005049 | chr21 | + | 44351635 | 44375491 | 5822 | NP_005040 | PWP2 periodic tryptophan protein homolog | chr21:44351384-44352241 | 1 |  | 1 | Promoter |
| PFKL | 1 |  | 1 | NM_002626 | chr21 | + | 44544357 | 44571684 | 5211 | NP_002617 | liver phosphofructokinase | chr21:44543943-44544992 | 1 |  | 1 | Promoter |
| SUMO3 |  | 1 | 1 | NM_006936 | chr21 | - | 45062472 | 45049959 | 6612 | NP_008867 | small ubiquitin-like modifier protein 3 | chr21:45061154-45063386 |  | 1 | 1 | Promoter |
| PTTG1IP | 1 |  | 1 | NM_004339 | chr21 | - | 45118169 | 45093940 | 754 | NP_004330 | pituitary tumor-transforming gene 1 | chr21:45117171-45119172 | 1 |  | 1 | Promoter |
| SLC19A1 |  | 1 | 1 | NM_194255 | chr21 | - | 45786779 | 45759056 | 6573 | NP_919231 | solute carrier family 19 member 1 | chr21:45786096-45787506 |  | 1 | 1 | Promoter |
| DIP2A |  | 1 | 1 | NM_015151 | chr21 | + | 46703289 | 46814354 | 23181 | NP_055966 | disco-interacting protein 2A isoform a | chr21:46702498-46704004 |  | 1 | 1 | Promoter |

**S_Table 1. The fifteen genes with aberrant methylation patterns located in chromosome 21**
